# Complete Prolapsus of Uterus

**Published:** 1899-02

**Authors:** J. S. Zvesper

**Affiliations:** Ammansville, Texas


### TEXAS MEDICAL JOURNAL.

ESTABLISHED JULY, 1885.

PUBLISHED MONTHLY.—SUBSCRIPTION $1.00 A YEAR.

VOL. XIV. AUSTIN, FEBRUARY, 1899. NO. 8.

### Original Contributions.

For Texas Medical Journal.

### Complete Prolapsus of Uterus.—Lacerated Perineum. A Comparatively New Operation Performed, with Report of Two Cases.

BY J. S. ZVESPER, M. D., AMMANSVILLE, TEXAS.

CASE I.—Mrs. C., white, 50 years of age, married, mother of ten living children; had several abortions; with a history of profuse uterine hemorrhage. She presented herself for treatment on July 1st, 1898, when, upon an examination, the following condition was discovered: Leucorrhœa profuse, uterus completely prolapsed and enormously swollen, resting between the thighs and much covered with ulcers, the result of friction and contact with the clothing, etc. This prolapsed condition of the uterus is said to have existed more than fourteen years, but grew much more troublesome during last four years. Upon reduction of same, which was done with good deal of pain, an old perineal laceration extending to the sphincter ani was found.

*Treatment:* Besides the constitutional treatment, strict local cleanliness, with copious hot water injections repeated several times during the day, were instituted, and continued until the engorgement had considerably decreased in size and the ulcerations nearly disappeared, when an operation was suggested, and consent being obtained, same was performed on July 15th, and another one two weeks later.

The primary operation consisted in radical curettement and the amputation of the cervix uteri in the ordinary way, silver sutures being selected, which were removed in two weeks, when the secondary operation for the reconstruction of the perineal floor was performed, which I wish to describe a little, as its technique materially differs from that usually practiced, and which proved admirably satisfactory. The patient having been prepared by all preliminary steps required for Emmet's operation, was anæsthetized by Dr. J. H. Adams, and placed in the lithotomy position. The left index finger was now introduced into the rectum, and a long, straight, double-edged bistoury made to pierce the tissuses in front of the anus at right angle to the vulva, and guided by the finger in the rectum made to penetrate the recto-vaginal septum for about two and one-half inches upwards, the incision being enlarged laterally to two inches as the bistoury was withdrawn.

This done, a strong, curved needle was introduced at lower edge of the incision, and, guided by the finger in rectum, was made to pass over the cut surface to its full depth above, and the point of the needle making its appearance directly opposite, it was threaded with strong silver suture and carried through. In this manner a sufficient number of sutures being introduced and properly adjusted, the two cut surfaces were brought into direct apposition. This then pushed the external tissues fully an inch and a half forward from anus, forming a thick and firm perineal body. This being over, the field of operation was cleansed and an antiseptic dressing applied, the legs being tied together to prevent the patient from unduly separating them, and placed in bed.

There was no fever after this operation, and the healing process was an uninterrupted one. Sutures were removed on the eighteenth day. In regard to bowels, I should add that they were moved on the fifth day by means of soap and water. The patient began to recover and was allowed to leave her bed and walk about in about six weeks from the time when primary operation was performed. Today we find the uterus very much reduced and well high up. There is no discomfort whatever, no leucorrhœa, and the patient very much pleased and happy.

The operation just now described is by no means my original one. I may have only deviated to some extent. The credit is due Dr. Alex. Duke, of Dublin, whose article I had read some eight years ago in some medical journal, while reading medicine at home, of which I made a memorandum in my note book. I consider this operation much easier than that by Dr. Emmet—usually performed —and in my humble estimation equally as good. Since there is no loss of tissue, there can be no harm done to those parts, and it always allows the other operation to be performed should this one just described fail. Furthermore, the secretions from uterus and vagina cannot come in contact with the incision, and consequently the wound is kept dry and clear, and there is no danger of non-union or sepsis. Finally, there is but little loss of blood, and it seems to be the very operation for weak and debilitated patients, whose state of health does not warrant us to undertake a more bloody and lengthy operation.

Case II.—Mrs. M. G., white, married, age 40 years, mother of several children, with a history of menorrhagia, leucorrhœa, etc. Had also one miscarriage, with uterus completely prolapsed, which condition lasted over twelve years. This case deserves no special description, as the treatment was as in the former case, with probably one exception, which is the fact that owing to her weakness, general anæsthesia was not deemed desirable, and local one only by means of chloride of ethyl and suggestion were made use of. This operation was also a success.

